# Sulforaphane’s Role in Osteosarcoma Treatment: A Systematic Review and Meta-Analysis of Preclinical Studies

**DOI:** 10.3390/biomedicines13051048

**Published:** 2025-04-25

**Authors:** Polymnia Louka, Nuno Ferreira, Antonia Sophocleous

**Affiliations:** 1 Translational Neuropharmacology Laboratory, Department of Psychology, University of Cyprus, Nicosia 2109, Cyprus; louka.polymnia@ucy.ac.cy; 2 Department of Social Sciences, University of Nicosia, 46, Makedonitissas Avenue, Engomi, Nicosia 2417, Cyprus; ferreira.n@unic.ac.cy; 3 Department of Life Sciences, School of Sciences, European University of Cyprus, 6, Diogenes Str., Engomi, Nicosia 2404, Cyprus

**Keywords:** sulforaphane, SFN, osteosarcoma, OSA, preclinical studies, systematic review, meta-analysis

## Abstract

**Background/Objectives**: Osteosarcoma (OSA) is the most common bone cancer, characterized by rapid progression and poor prognosis. The isothiocyanate sulforaphane (SFN), has gained scientific interest because of its potent anticancer properties. The aim of this study was to conduct a systematic review of research examining the effectiveness of SFN as a treatment for OSA. **Methods**: A literature search was conducted using MEDLINE, EMBASE, and Web of Science. Studies evaluating the therapeutic efficacy of SFN on OSA were included, while studies examining the effects of isothiocyanates other than SFN were excluded. The quality of the studies was evaluated using the OHAT risk of bias rating tool, and the meta-analysis was conducted using RevMan. Cancer-related outcomes evaluated included cell viability/migration/invasion, cell cycle arrest, apoptosis induction, antioxidant activity, colony formation, and tumour size. A protocol describing the review plan was registered to INPLASY (INPLASY202530001). **Results:** Ten articles were considered eligible for qualitative synthesis and meta-analysis. All articles included in vitro studies, with two also incorporating in vivo studies, utilizing a combination of human, canine, and murine OSA cell lines. This review indicates that SFN could be beneficial in the treatment of OSA, particularly by reducing cell viability, inducing apoptosis, arresting the cell cycle, and decreasing invasiveness and migration. It emphasizes dose-dependent effects, the need for human trials, and highlights limitations like study heterogeneity and SFN’s bioavailability challenges. **Conclusions:** This review explores SFN’s potential in OSA at the preclinical stage, focusing on cell apoptosis and proliferation. It highlights promising evidence but calls for more human trials. This research received no external funding.

## 1. Introduction

Osteosarcoma (OSA) is a type of malignant, primary bone tumour that is associated with early metastasis, quick progression, and poor prognosis [1]. OSA is the most commonly reported form of bone cancer in children and young adults (mostly 14–18 years of age) and can occur in any bone [2]. The most common sites of occurrence are the femur (42%), the tibia (19%) and the humerus (10%) as OSA is most likely to present near the metaphyseal growth plates of the long bones [3]. Management of newly diagnosed OSA usually involves initial neoadjuvant chemotherapy followed by the surgical removal of the tumour and metastatic spreads, and lastly adjuvant chemotherapy for the elimination of any remaining growths [4]. With a 5-year survival rate of approximately 63%, OSA still presents very high mortality when cases are detected at later stages [5], therefore there is still a need for new antitumour agents or chemicals to be developed.

As in many other cancers, the molecular pathogenesis of OSA still remains unclear, however, studies suggest a disruption in the cell cycle and apoptosis [6]. One family of chemical compounds that are known to regulate these processes are isothiocyanates (ITCs) which result from the hydrolysis of glucosinolates [7]. Sulforaphane (SFN), a naturally occurring isothiocyanate found in cruciferous vegetables like broccoli, has therefore emerged as a promising intervention for OSA.

**Figure 1:**
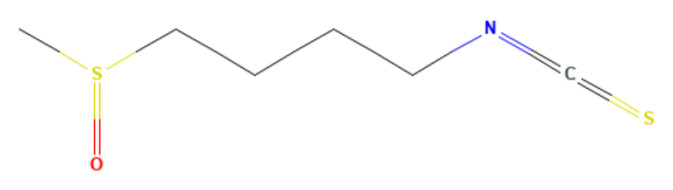
Chemical structure of sulforaphane. PubChem, CID 5350, Sulforaphane. https://pubchem.ncbi.nlm.nih.gov/compound/Sulforaphane (accessed on 26 March 2025) [8].

SFN has a chemical structure of 4-methylsulfinylbutyl isothiocyanate or 1-isothiocyanate-4-methylsulfinylbutane (Figure 1) with a molecular formula of C_6_H_11_S_2_NO and a relative molecular weight of 177.3 g/mol [8]. SFN is a phytochemical that occurs in plants in the form of a biologically inactive precursor glucoraphanin [9].

Its potential anti-cancer effects are exerted through multiple mechanisms that intersect with the pathways driving OSA development. A recent review [10] indicates that SFN can induce apoptosis in cancer cells by activating the mitochondrial apoptotic pathway and enhancing the expression of death receptor 5 (DR5), which sensitizes cells to TNF-related apoptosis-inducing ligand (TRAIL)-induced cell death. Additionally, SFN is known to cause cell cycle arrest in the G2/M phase, impeding the proliferation of cancer cells. It can inhibit aberrant signaling through the PI_3_K/AKT and MAPK pathways, pathways integral to OSA progression, by modulating key signaling molecules and reducing phosphorylation events. Furthermore, SFN has been noted to downregulate angiogenic factors and matrix metalloproteinases, thereby restraining tumour vascularization and metastatic potential. It is important to notice that these effects have been observed in various cancer cells such as those affecting the bladder, brain, breast, colorectal, lung, ovarian, pancreatic, prostate, and skin [10].

While no clinical trials have specifically studied SFN in OSA patients, it has shown promising results in various other cancers (reviewed in [11]). In prostate cancer, SFN reduced disease progression and severity. For melanoma, it lowered plasma proinflammatory cytokines and boosted the tumour suppressor decorin. In recurrent prostate cancer, SFN did not significantly reduce PSA levels but was safe and showed positive effects on PSA Doubling Time (PSADT), which correlates with survival. In low- or intermediate-grade prostate cancer, SFN influenced enzyme expression and altered levels of key biomarkers, including F2 isoprostane and 8-hydroxy-deoxyguanosine (8OHdG) for oxidative stress, dihydrotestosterone, and testosterone, suggesting potential therapeutic benefits.

When combined with other anticancer treatments, preclinical studies have shown that SFN exerts synergistic anticancer effects and helps to reduce adverse effects (reviewed in [12]). For colorectal cancer, SFN with Salinomycin leads to reduced cell migration and invasion, increases apoptosis, and inhibits proliferation, while in breast cancer, SFN with Biochanin A and Withaferin A induces apoptosis and inhibits cell cycle progression. SFN and Fernblock^®^ extract combination inhibited the production of matrix metalloproteinases in melanoma cells and reduced melanoma cell migration. SFN enhances cisplatin sensitivity in ovarian cancer, while in cholangiocarcinoma, SFN works synergistically with cisplatin to promote apoptosis. Finally, in glioblastoma, SFN with the peptide nucleic acid, PNA-a15b, increases cellular apoptosis.

These multifaceted actions of SFN highlight its potential as a therapeutic agent targeting various pathways involved in tumour progression, but results seem to vary depending on dose, cancer type studied, and experimental design.

While promising preclinical data exist, the bioavailability and effective dosing of SFN in vivo remain underexplored. Additionally, there is a notable gap in clinical trials evaluating the safety, efficacy, and long-term outcomes of SFN in OSA patients. By focusing on preclinical studies, this systematic review and meta-analysis aims to consolidate current findings on SFN’s effects on OSA, explore the use of different SFN concentrations that successfully disrupt pathways related to OSA development, and thereby provide a strong foundation for future clinical trials assessing new therapies for OSA patients.

## 2. Materials and Methods

The Preferred Reporting Items for Systematic Review and Meta-Analysis (PRISMA) criteria were used for conducting and reporting the results of this systematic review [13]. The protocol of the present review has been registered with the International Platform of Registered Systematic Review and Meta-Analysis Protocols (INPLASY) and its registration number is INPLASY202530001 (full protocol can be accessed here: https://inplasy.com/inplasy-2025-3-0001/ [accessed on 4 March 2025]). For the PRISMA 2020 Checklist and the PRISMA 2020 for Abstract Checklist, please see Tables S1 and S2, respectively, in the Supplementary Materials.

### 2.1. Search Strategy

The following three databases were used to perform a systematic search on 26 February 2024; Medline [Ovid MEDLINE(R) ALL <1946 to 26 February 2024>], EMBASE (Embase Classic + Embase <1947 to 2024 Week 09>), and Web of Science (WOS: 1900 to 2024). An updated search was performed on 6 April 2025 to capture any newly published studies since the original search. Medical subject heading (MeSH) keywords and free words, along with their synonyms, were used to search each database for the concepts “osteosarcoma” and “sulforaphane”, in conjunction with the Boolean logic operation “OR”/“AND”. Detailed search strategies are provided in Supplementary Tables S3 and S4.

### 2.2. Inclusion/Exclusion Criteria and Study Selection

**Figure 2:**
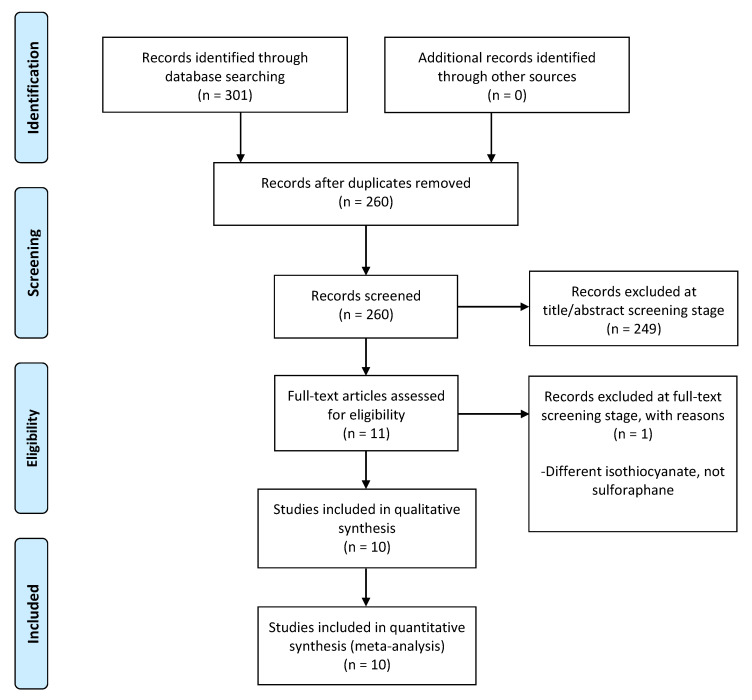
Prisma flow diagram.

Preclinical studies were included in this systematic review and meta-analysis. Studies that used in vitro and in vivo models to assess the effects of SFN on cell viability, cell cycle distribution, apoptosis induction, antioxidant activity, colony formation, cell migration/invasion, and tumour size were included. Exclusion criteria included reviews, commentaries, and editorial pieces; studies published in a language other than English; and studies that reported the effects of isothiocyanates other than SFN. Initially, all duplicate studies were identified and removed. Screening was performed by P.L. and A.S., at two stages of review: title/abstract and full text. Titles and abstracts were checked against the inclusion and exclusion criteria, and if a conflict arose, it was resolved by discussion or referral to a third reviewer (N.F.). The PRISMA flow chart of the included studies’ strategies is presented graphically in Figure 2.

### 2.3. Outcomes Measures

The outcome measures chosen to assess the anticancer efficacy of SFN in the present systematic review and meta-analysis were as follows: the measurement of cell growth (by the MTT and CCK-8 assays, as well as by the trypan blue dye exclusion test); analysis of cell cycle progression (using flow cytometry); the % of apoptotic cells and measurement of Caspase-3 activity; the intracellular glutathione and reactive oxygen species (ROS) levels, as well as the measurement of total antioxidant activity, number of colonies formed, number of invading cells, and measurement of cell mobility/migration (by the wound healing assay); and finally, tumour volume from in vivo experiments.

### 2.4. Data Items and Data Extraction

Data extracted included study details (authors, year of publication, cell lines, intervention, anticancer outcomes assessed, study results, and funding source). For studies including in vivo experiments, additional experimental design details were extracted (species, sex, sample size, age, and treatment regimen). Data for each outcome following treatment with SFN or vehicle/control were also collected. Mean and standard deviation (SD) or standard error of the mean (SEM) were extracted from relevant tables or figures using the online tool WebPlotDigitizer Version 4.8 (https://apps.automeris.io/wpd4/) (accessed on 1 July 2024 to 13 April 2025) [14]. Briefly, WebPlotDigitizer extracts data from graphs by calibrating axes, clicking on graph points, and exporting the data into spreadsheets. A step-by-step breakdown of how WebPlotDigitizer works is shown in Table S5.

For studies that reported mean ± SEM, SD was calculated using the following formula: SEM = SD/sqrt(N). If neither variance nor SEM were provided, SD was imputed from the weighted average of variances observed in other studies as previously described [15,16]. In particular, the SD of the G0/G1, S and G2/M phases following the treatment of MG-63 cells with 20 μM SFN for 24 h, reported in the Matsui et al., 2007 study [17], was imputed from weighted average of variances of the G0/G1, S and G2/M phases, respectively, following the treatment of the same cells (MG-63), with the same concentration of SFN (20 μM) and for the same period of time (24 h), reported in the studies Kim et al., 2011 [18] and Ferreira de Oliveira et al., 2014b [7]. Similarly, the SD of the Sub-G1 phase following the treatment of MG-63 cells with 20 μM SFN for 24 h, reported in the Matsui et al., 2006 study [19], was imputed from weighted average of variances of the Sub-G1 phase of LM8 cells following the treatment with the same concentration of SFN (20 μM) and for the same period of time (24 h), reported in the studies Matsui et al., 2007 [17] and Sawai et al., 2013 [20]. All data were extracted independently by a reviewer (P.L.) and a second reviewer verified the data extraction (A.S.). Consensus over discrepancies was reached through discussion or in consultation with a third reviewer (N.F.).

### 2.5. Data Analysis

Data from the included studies were analyzed from 4 November 2024 through 9 December 2024. Meta-analysis was performed using Review Manager (RevMan) version 5.3 [21] if at least 2 studies deemed of the same design were identified. The mean difference was used as the effect measure if the same outcome and unit of measure were used in all studies included in a forest plot. Otherwise, standardized (std.) mean difference was used as the effect measure. Mean different and std. mean difference were calculated using the inverse-variance method and the fixed effect analysis model was used when heterogeneity was considered small to moderate (i.e., I^2^ < 50%). If heterogeneity was high (i.e., I^2^ > 50%), then the random effect analysis model was used. The 95% confidence intervals (95% CI), *p* value, and the test for overall effects (z-value) were calculated and forest plots were generated. All analyses were carried out by entering the mean, standard deviation, and number of repetitions performed (usually experiments were repeated three times). If an outcome was not reported uniformly in at least 2 studies, then meta-analysis was not possible, and narrative synthesis was conducted. To increase transparency and rigour of the process of conducting narrative synthesis, data were checked and interpretations were agreed upon by two reviewers (P.L. and A.S.).

### 2.6. Quality Review

Quality and bias review was assessed by A.S. and P.L., using the OHAT (Office of Health Assessment and Translation) risk of bias rating tool [22] for in vitro studies. The OHAT risk of bias tool assesses possible sources of bias arising from randomization, allocation concealment, experimental conditions, blinding, complete outcome data, exposure characterization, outcome assessment, outcome reporting, and no other threats [22]. Any disagreement when using the quality assessment tool was resolved by consensus.

### 2.7. Certainty of Evidence

Certainty of the evidence for in vivo and in vitro outcomes was assessed using the grading of recommendations assessment, development, and evaluation (GRADE) approach, adapted for preclinical systematic reviews [23,24]. For data that were in a format unsuitable for pooling in a single estimate of effect through meta-analysis, and instead were summarized narratively (narrative synthesis), the constructs of the GRADE approach were applied once again, while using the additional guidance provided by Murad and colleagues, 2017 [25]. Upgrading was considered relevant where there was a dose–response relationship [24].

### 2.8. Publication Bias

It was not possible to assess for publication bias using the funnel-plot asymmetry test, because none of the pooled analyses included 10 or more studies, and according to the Cochrane Handbook for Systematic Reviews of Interventions (version 5.2.0) [26], the power of the test in these instances would be too low to distinguish chance from real asymmetry. Therefore, any interpretation should be carried out with a degree of caution.

## 3. Results

### 3.1. Study Selection

A total of 301 records were identified through the database searches and 41 duplicate records were removed. Out of the remaining 260 records, 249 were excluded based on their title and/or abstract. At this stage, articles were excluded primarily due to irrelevance, or because they focused on an isothiocyanate other than sulforaphane, or on a cancer type other than osteosarcoma. In total, 11 full-text records were screened for the inclusion criteria and 10 of them were considered to be eligible for the qualitative and quantitative synthesis (meta-analysis). Details of the study excluded in the full-text stage can be found in Supplementary Table S6. The PRISMA flow diagram in Figure 2 summarises the study selection process.

### 3.2. Study Characteristics

All 10 articles included were published from 2006 to 2025 and featured in vitro studies. At least one in vitro study from each selected article was included in the quantitative synthesis (meta-analysis). Some of the data of 7 out of 10 articles were reported in a format unsuitable for pooling or considered too heterogeneous and were therefore included in narrative synthesis [17,19,27,28,29,30,31]. Out of the 10 included articles, 2 also included in vivo studies [17,31]. Two researchers independently reviewed each article and identified relevant studies in each article included. The main characteristics of the included studies are summarised in Table 1.

### 3.3. Quality Assessment

**Figure A1:**
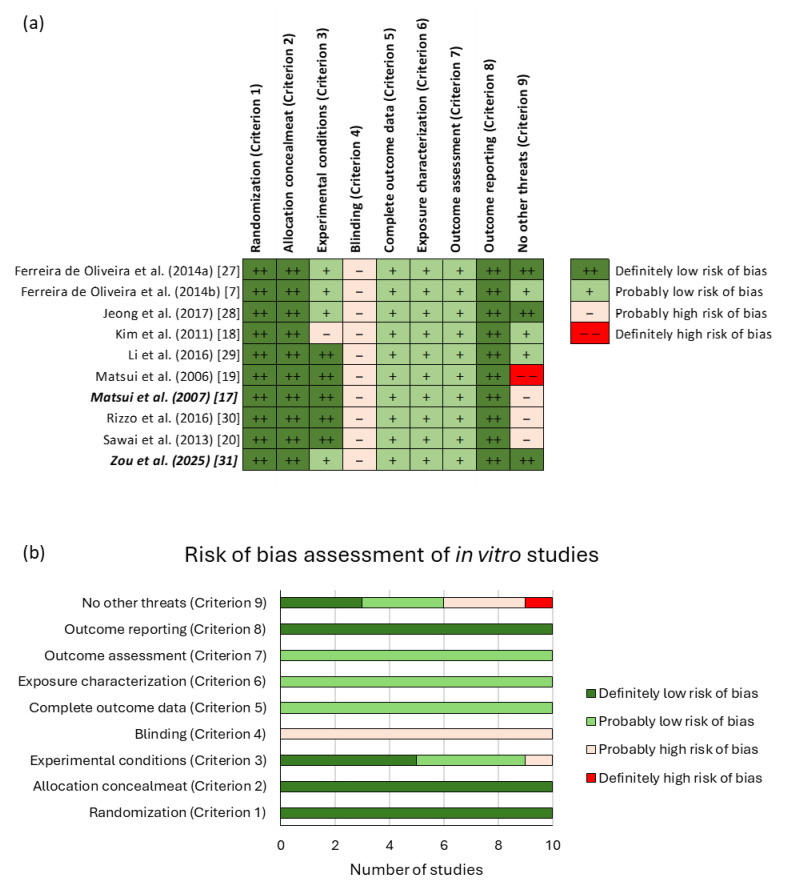
Risk of bias (RoB) assessment for included studies using the OHAT RoB tool. (**a**) Representative summary Table for the risk of bias assessment. Dark green cells with ‘++’ designate definitely low risk of bias, light green cells with ‘+’ designate low risk of bias, light pink cells with ‘−’ designate probably high risk of bias, and red cells with ‘− −’ designate definitely high risk of bias. (**b**) Representative summary of risk of bias analysis across studies. In bold and italics are the articles that consist of in vitro and in vivo experiments.

The OHAT risk of bias rating tool [22] was used to assess the risk of bias of included studies (Appendix A, Figure A1). Out of the 9 criteria, only ‘Blinding of research personnel during the study’ (criterion 4) was scored as ‘Probably high risk’ across all in vitro studies. This was of no concern, however, since it is rather uncommon for researchers to be blinded when performing in vitro experiments. For all remaining criteria, apart from criterion 3 (Experimental conditions) and criterion 9 (No other threats), all studies were considered ‘Definitely low risk’ or ‘Probably low risk’. For criterion 3 (Experimental conditions), 1 article was considered ‘Probably high risk’. For criterion 9 (No other threats), 3 studies were considered ‘Probably high risk’, because of inappropriate statistical methods and/or because no statement regarding conflict of interest was provided; 1 study was considered ‘Definitely high risk’ because graphs lacked error bars and no statistical methods were reported. The overall risk of bias for 9 out of 10 in vitro studies was ‘Probably low’ to ‘Definitely low’ (i.e., Tier 1 on a 3-tier system), and the overall risk of bias for the 10th study did not meet the criteria either for ‘low’ or ‘high’ (i.e., Tier 2 on a 3-tier system). Because the overall risk of bias of none of the studies was considered ‘Definitely high’ or ‘Probably high’ risk of bias (i.e., Tier 3 on a 3-tier system), no study was excluded solely based on their quality.

### 3.4. Assessment of Certainty of Evidence

The overall quality of outcomes of in vitro and in vivo studies was judged to be (A) low for in vitro cell viability/invasion, apoptosis indices, and colony number and in vivo tumour volume/weight, and (B) very low for in vitro cell migration/cycle distribution and antioxidant activity. The effect of SFN on the above-mentioned outcomes was rather limited because these studies are preclinical studies (i.e., of no randomized controlled clinical trial study design), and because of moderate to high heterogeneity amongst studies, suggesting inconsistency. However, given the dose-dependent effects of the SFN observed on cell viability and invasion, apoptosis indices, colony number, and tumour volume and weight, the evidence for these outcomes was upgraded by one level, from a very low to a low certainty of evidence.

### 3.5. Meta-Analysis Interpretation and Narrative Synthesis

The study outcomes are summarized in forest plots, presenting significant improvement or worsening of cancer-relevant outcomes following treatment with SFN. Relevant narrative synthesis of data that were unsuitable to be pooled in the meta-analysis is also included in each of the following sections.

#### 3.5.1. Effect of SFN on Cell Viability

**Figure 3:**
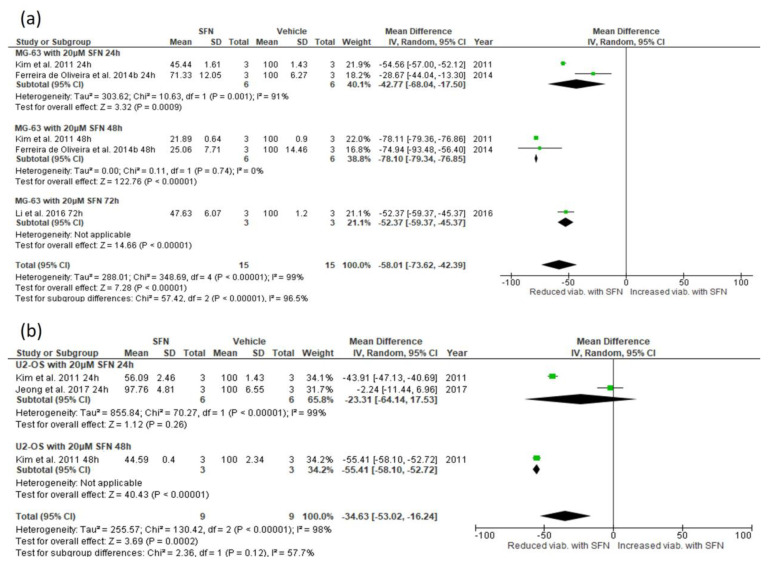
Forest plots showing the effect of 20 μΜ SFN on the viability of (**a**) MG-63 and (**b**) U-2 OS cells. Data from the included studies were categorized into subgroups based on the duration of the 20 μM SFN treatment. The green squares represent the estimated mean difference for each individual study and the black diamond represents the pooled mean difference and the associated 95% CI. The studies included in these forest plots are: Ferreira de Oliveira et al. (2014b) [7], Kim et al. (2011) [18], Jeong et al. (2017) [28], Li et al. (2016) [29].

Treatment with 20 μΜ SFN significantly reduced MG-63 cell viability after 72, 48, and 24 h treatment (72 h: mean difference −52.37, 95% CI −59.37, −45.37, *p* < 0.001; 48 h: mean difference −78.09, 95% CI −79.34, −76.85, *p* < 0.001; 24 h: mean difference −42.77, 95% CI −68.03, −17.51, *p* < 0.001) [7,18,28] (Figure 3a). Collectively, treatment of MG-63 cells with 20 μΜ SFN significantly reduced cell viability, with a Z value of 7.28, which corresponds to a *p* < 0.00001 (mean difference −58.01, 95% CI −73.62, −42.39). Treatment of MG-63 cells with 10 μΜ SFN also significantly reduced cell viability, with a Z value of 2.53 (*p* = 0.01) and mean difference −41.40 (95% CI −73.50, −9.31). The concentration of 5 μΜ SFN, however, did not significantly reduce MG-63 cell viability (Z = 1.87, *p* = 0.06; mean difference −26.05, 95% CI −53.30, 1.19) (Appendix B, Table A1).

In the U-2 OS cell line, treatment with 20 μΜ SFN significantly reduced cell viability after 48 h treatment (mean difference −55.41, 95% CI −58.09, −52.72, *p* < 0.001), but not after 24 h treatment (mean difference −23.30, 95% CI −64.13, 17.52, *p* = 0.26) [18,28] (Figure 3b). Collectively, however, treatment of U-2 OS cells with 20 μΜ SFN significantly reduced cell viability, with a Z value of 3.69, which corresponds to a *p* = 0.0002 (mean difference −34.62, 95% CI −53.01, −16.24). Although a concentration of 10 μΜ SFN did not significantly reduce U-2 OS cell viability (Z = 1.23, *p* = 0.22; mean difference −14.76, 95% CI −38.23, 8.71), a concentration of 5 μΜ SFN did significantly reduce U-2 OS cell viability, with a Z value of 3.01 (*p* = 0.003) and mean difference −7.95 (95% CI −13.12, −2.78) (Appendix B, Table A1).

#### 3.5.2. Narrative Synthesis for the Effect of SFN on Cell Viability and Colony Number

Data from other OSA cell lines showed that treatment with 5–20 μΜ SFN significantly reduced the cell viability of the human cell lines OS-732 [29], 143B [31], and SJSA-1 [31], and treatment with 6.25–25 μΜ SFN significantly reduced the cell viability of the canine cell line HMPOS [30] (Appendix C, Table A2). Other canine cell lines, however, responded differently. A small concentration of SFN (6.25 μΜ) significantly increased the cell viability of D17 and OS 2.4 canine cell lines, but a 4-fold increase in SFN concentration (25 μΜ) significantly reduced the cell viability of both cell lines (Appendix C, Table A2).

Data on colony formation showed that 1.25–5μM SFN significantly reduced the number of colonies formed in the human cell lines 143B and SJSA-1, in a dose-dependent manner [31] (Appendix C, Table A2).

#### 3.5.3. Effect of SFN on Cell Cycle Distribution

**Figure A2:**
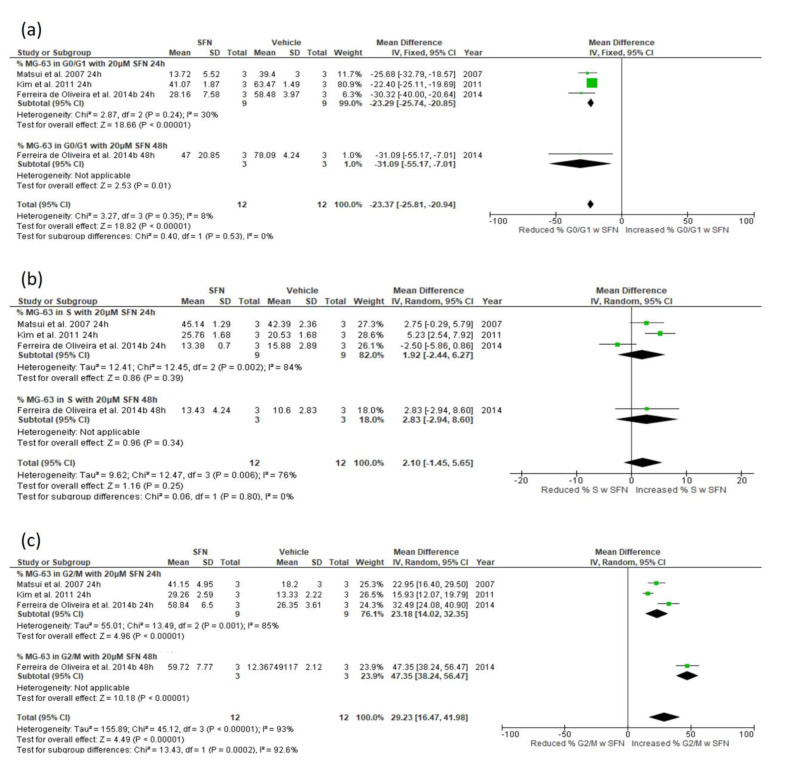
Forest plots showing the effect of 20 μΜ SFN on the distribution of MG-63 cells in (**a**) G0/G1, (**b**) S, and (**c**) G2/M phases of the cell cycle. Data from the included studies were categorized into subgroups based on the duration of the 20 μM SFN treatment. The green squares represent the estimated mean difference for each individual study and the black diamond represents the pooled mean difference and the associated 95% CI. The studies included in these forest plots are: Ferreira de Oliveira et al. (2014b) [7], Matsui et al. (2007) [17], Kim et al. (2011) [18].

Treatment with 20 μΜ SFN significantly reduced the fraction of MG-63 cells in G0/G1 phase after 48 and 24 h treatment (48 h: mean difference −31.10, 95% CI −55.17, −7.02, *p* = 0.01; 24 h: mean difference −23.29, 95% CI −25.74, −20.85, *p* < 0.001) (Appendix A, Figure A2a). Collectively, treatment of MG-63 cells with 20 μΜ SFN significantly reduced the G0/G1 phase of the cell cycle, with a Z value of 18.82, which corresponds to a *p* < 0.001 (mean difference −23.37, 95% CI −25.81, −20.94) (Appendix A, Figure A2a).

Treatment with 20 μΜ SFN did not significantly alter the fraction of MG-63 cells in S phase after 24 or 48 h treatment (24 h: mean difference 1.91, 95% CI −2.46, 6.28, *p* = 0.39; 48 h: mean difference 2.83, 95% CI −2.94, 8.59, *p* = 0.34) (Appendix A, Figure A2b). Collectively, treatment of MG-63 cells with 20 μΜ SFN did not have an impact on the S phase of the cell cycle, since the overall effect Z value is 1.15, which corresponds to a *p* value of 0.25 (mean difference 2.09, 95% CI −1.48, 5.65) (Appendix A, Figure A2b).

Treatment with 20 μΜ SFN significantly increased the fraction of MG-63 cells in G2/M phase after 24 and 48 h treatment (24 h: mean difference 23.18, 95% CI 14.01, 32.35, *p* < 0.001, 48 h: mean difference 47.35, 95% CI 38.23, 56.47, *p* < 0.001) (Appendix A, Figure A2c). Collectively, treatment of MG-63 cells with 20 μΜ SFN significantly increased the G2/M phase, with a Z value of 4.49, which corresponds to a *p* < 0.001 (mean difference 29.22, 95% CI 16.47, 41.97) (Appendix A, Figure A2c).

Treatment of MG-63 cells with smaller concentrations of SFN, namely 5 μΜ and 10 μΜ, also significantly reduced the population of cells in G0/G1 phase of the cell cycle after 24 and 48 h treatment (5 μM: Z = 3.10, *p* = 0.002, mean difference −10.39, 95% CI −16.96, −3.83; 10 μM: Z = 12.94, *p* < 0.00001, mean difference −20.11, 95% CI −23.15, −17.06) (Table A1). No significant difference in the fraction of MG-63 cells in the S phase was observed, up to 48 h following treatment with either 5 or 10 μM SFN (5 μM: Z = 0.64, *p* = 0.52, mean difference 2.54, 95% CI −5.19, 10.26; 10 μM: Z = 0.31, *p* = 0.76, mean difference 0.93, 95% CI −5.03, 6.89) (Table A1). However, the population of MG-63 in the G2/M phase under the same conditions was significantly increased (5 μM: Z = 5.76, *p* < 0.00001, mean difference 10.36, 95% CI −6.84, 13.89; 10 μM: Z = 9.22, *p* < 0.00001, mean difference 16.98, 95% CI 13.37, 20.59) (Table A1). Collectively, these results indicate that SFN causes G_2_/M phase cell cycle arrest in MG-63 cells.

#### 3.5.4. Narrative Synthesis for the Effect of SFN on Cell Cycle Distribution

A significant increase in the fraction of cells in the G2/M phase following a 24 or a 48 h treatment with 20 μΜ SFN was also evident in the canine OSA cell lines, D17, OS 2.4, and HMPOS [30], and in the murine OSA cell line LM8 [17], respectively (Appendix C, Table A3). However, this was not mirrored in the human OSA cell lines, OS-732 and MG-63, following a 72 h treatment with 15 μΜ SFN [29] (Appendix C, Table A3).

#### 3.5.5. Effect of SFN on Apoptosis Indices

**Figure A3:**
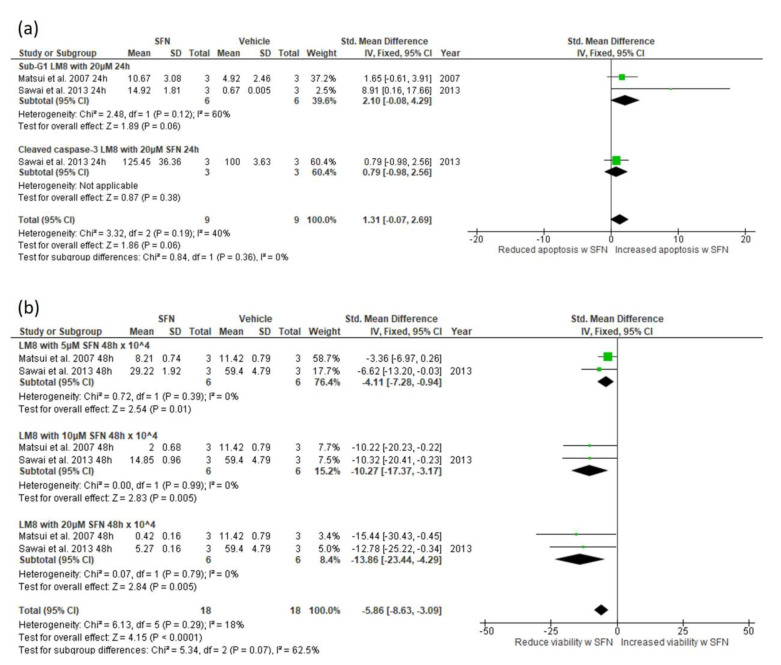
Forest plots showing the effect of 5–20 μΜ SFN on apoptosis indices in LM8 cells. (**a**) Distribution of LM8 cells in sub-G1 phase and active caspase-3 levels in LM8 cells after 24 h of treatment. Data from the included studies were categorized into subgroups based on the apoptosis indicator. (**b**) LM8 cell viability after 48 h treatment, as shown by the trypan blue dye exclusion test. Data from the included studies were categorized into subgroups based on the concentration of the SFN treatment. The green squares represent the estimated std. mean difference for each individual study and the black diamond represents the pooled std. mean difference and the associated 95% CI. The studies included in these forest plots are: Matsui et al. (2007) [17], Sawai et al. (2013) [20].

Treatment with 20 μΜ SFN did not significantly alter the fraction of LM8 cells in the sub-G1 phase or the active caspase-3 levels in LM8 cells after 24 h of treatment (Sub-G1 levels: std. mean difference 2.10, 95% CI −0.08, 4.29, *p* = 0.06; Cleaved caspase-3 levels: std. mean difference 0.79, 95% CI −0.98, 2.56, *p* = 0.38) (Appendix A, Figure A3a). Collectively, treatment of LM8 cells with 20 μΜ SFN did not have an impact on apoptosis indices, since the overall effect Z value is 1.86, which corresponds to a *p* value of 0.06 (std. mean difference 1.31, 95% CI −0.07, 2.69) (Appendix A, Figure A3a).

Treatment of MG-63 cells with 20 μΜ SFN also did not significantly alter the fraction of cells in the sub-G1 phase or the active caspase-3 levels after 24 and 48 h of treatment, respectively (Z = 1.78, *p* = 0.07, mean difference 2.94, 95% CI −0.30, 6.18) (Table A1).

The trypan blue dye exclusion test, however, showed that treatment with 5, 10, and 20 μΜ SFN significantly reduced the viability of murine LM8 cells after 48 h treatment (5 μΜ: std. mean difference −4.11 95% CI −7.28, −0.94, *p* = 0.01; 10 μΜ: std. mean difference −10.27, 95% CI −17.37, −3.17, *p* = 0.005; 20 μΜ: std. mean difference −13.86, 95% CI −23.44, −4.29, *p* = 0.005). Collectively, treatment of LM8 cells with 5, 10, and 20 μΜ SFN for 48 h significantly reduced the number of viable cells, with a Z value of 4.15, which corresponds to a *p* < 0.001 (std. mean difference −5.86, 95% CI −8.63, −3.09) (Appendix A, Figure A3b). This was also the case for LM8 cells treated with 5, 10, and 20 μΜ SFN for the period of 24 h (Z = 3.87, *p* = 0.0001, mean difference −4.30, 95% CI −6.47, −2.12) (Table A1).

#### 3.5.6. Narrative Synthesis for the Effect of SFN on Apoptosis Indices

Treatment of LM8 cells with concentrations of SFN ranging from 2.5 to 15 μΜ, did not significantly alter the population of cells in the sub-G1 phase of the cell cycle after 24 h treatment (Appendix C, Table A4).

The trypan blue dye exclusion test by Matsui and colleagues (2007) [17], however, showed that the percentage of viable MG-63 cells was significantly reduced following a 24 h treatment with 10 and 20 μΜ SFN. For twice as long treatment periods, i.e., 48 h, the viability of MG-63 was significantly reduced with SFN concentrations as low as 5 μΜ (Appendix C, Table A5). Despite this, the trypan blue exclusion test failed to prove that SFN could induce cell death at concentrations up to 20 μΜ in three canine OSA cell lines, namely D17, OS 2.4, and HMPOS (Appendix C, Table A5).

#### 3.5.7. Effect of SFN on Percentage of Apoptotic Cells

**Figure 4:**
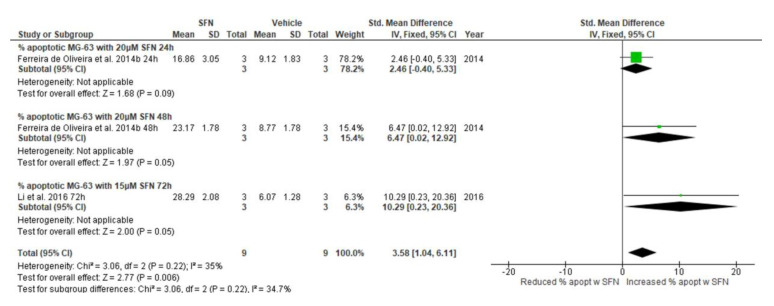
Forest plots showing the effect of 20 and 15 μΜ SFN on the percentage of apoptotic MG-63 cells. Data from the included studies were categorized into subgroups based on the duration of the SFN treatment. The green squares represent the estimated std. mean difference for each individual study and the black diamond represents the pooled std. mean difference and the associated 95% CI. The studies included in this forest plot are: Ferreira de Oliveira et al. (2014b) [7], Li et al. (2016) [29].

Treatment with 15 and 20 μΜ SFN significantly increased the percentage of apoptotic MG-63 cells after 48 and 72 h treatment, respectively (20 μΜ: standardized [std.] mean difference 6.47, 95% CI 0.02, 12.92, *p* = 0.05; 15 μΜ: std. mean difference 10.29, 95% CI 0.23, 20.36, *p* = 0.05). However, 24 h treatment of MG-63 cells with 20 μΜ SFN, did not have an impact on the percentage of apoptotic MG-63 cells. Collectively, treatment of MG-63 cells with 15 and 20 μΜ SFN for 24, 48, and 72 h significantly increased the percentage of apoptotic cells, with a Z value of 2.77, which corresponds to a *p* = 0.006 (std. mean difference 3.58, 95% CI 1.04, 6.11) (Figure 4).

#### 3.5.8. Narrative Synthesis for the Effect of SFN on the Percentage of Apoptotic Cells

Smaller SFN concentrations (5 and 10 μΜ) also increased the percentage of apoptotic MG-63 cells, but to a lesser extent (Appendix C, Table A6). The percentage of apoptotic cells was also significantly increased in the human OSA cell line OS-732 treated with 15 μΜ SFN for 72 h (Appendix C, Table A6).

#### 3.5.9. Effect of SFN on Antioxidant Indices

**Figure 5:**
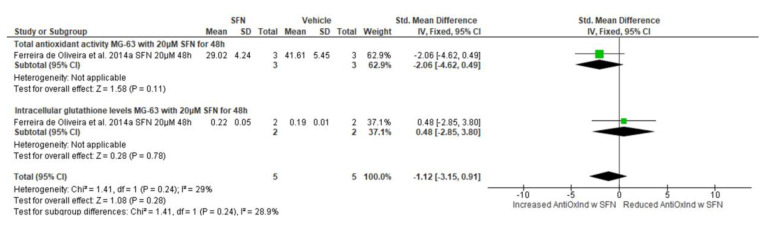
Forest plot showing the effect of 20 μΜ SFN on antioxidant indices (AntiOxInd: antioxidant activity and glutathione) in MG-63 cells. Data from the included studies were categorized into subgroups based on the antioxidant indicator. The green squares represent the estimated std. mean difference for each individual study and the black diamond represents the pooled std. mean difference and the associated 95% CI. The studies included in this forest plot are both from Ferreira de Oliveira et al. (2014a) [27].

Treatment with 20 μΜ SFN did not significantly alter the total antioxidant activity or the intracellular glutathione levels of MG-63 after 48 h of treatment (total antioxidant activity: standardized [std.] mean difference −2.06, 95% CI −4.62, 0.49, *p* = 0.11; intracellular glutathione levels: std. mean difference 0.48, 95% CI −2.85, 3.80, *p* = 0.78) (Figure 5). Collectively, treatment of MG-63 cells with 20 μΜ SFN for 48 h did not change antioxidant indices, since the overall effect Z value is 1.08, which corresponds to a *p* value of 0.28 (std. mean difference −1.12, 95% CI −3.15, 0.91).

Treatment of MG-63 cells with smaller concentrations of SFN, namely 5 μΜ and 10 μΜ, also did not significantly alter the total antioxidant activity or the intracellular glutathione levels after a 48 h period (5 μM: Z = 0.18, *p* = 0.86, std. mean difference −0.14, 95% CI −1.74, 1.45; 10 μM: Z = 0.21, *p* = 0.84, std. mean difference −0.17, 95% CI −1.77, 1.43) (Table A1). Likewise, no significant changes were observed in the total antioxidant activity or the intracellular glutathione levels in MG-63 cells treated with 5, 10, or 20 μM SFN for shorter periods of time, i.e., 24 h (5 μM: Z = 0.75, *p* = 0.45, std. mean difference −0.65, 95% CI −2.33, 1.04; 10 μM: Z = 0.87, *p* = 0.38, std. mean difference 0.66, 95% CI −2.15, 0.83; 20 μM: Z = 1.82, *p* = 0.07, std. mean difference −3.15, 95% CI −6.53, 0.24) (Table A1).

#### 3.5.10. Narrative Synthesis for the Effect of SFN on Reactive Oxygen Species Formation

According to Ferreira de Oliveira and colleagues, 2014a [27], SFN treatment for 24 and 48 h induced ROS accumulation in MG-63, in a concentration-dependent manner (Appendix C, Table A7). Similarly, Zou and colleagues, 2025 [31], showed that SFN increased the relative ROS formation in the human cell lines, 143B and SJSA-1 (Appendix C, Table A7). This was mirrored in the canine cell line OS 2.4 and to a lesser extent in the D17 cell line, but not in the HMPOS OSA cell line, where, instead, a 7-fold decrease in ROS accumulation was recorded following treatment with 20 μΜ SFN (Appendix C, Table A7).

#### 3.5.11. Effect of SFN on Cell Invasion and Cell Migration

**Figure 6:**
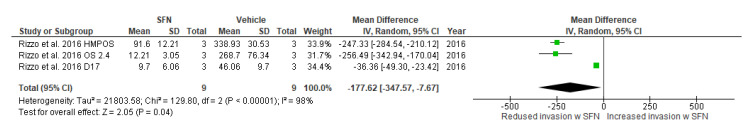
Forest plot showing the effect of 20 μΜ SFN on the number of invaded cells in three canine OSA cell lines, including HMPOS, OS 2.4, and D17. The studies included in this forest plots are all from Rizzo et al. (2016) [30].

A 24 h treatment with 20 μΜ SFN significantly reduced the invasive abilities of the canine OSA cells HMPOS, OS 2.4, and D17, with an overall Z value of 2.05, which corresponds to a *p* = 0.04 (mean difference −177.62, 95% CI −347.57, −7.67) (Figure 6).

Treatment of the above-mentioned osteosarcoma cell lines with a smaller concentration of SFN (10 μΜ) also significantly reduced the number of invaded cells after a 24 h treatment, albeit to a lesser extent (Z = 2.94, *p* = 0.003, mean difference −25.21, 95% CI −42.01, −8.42) (Table A1). Overall, the invasive abilities of these cells seemed to be attenuated by SFN in a dose-dependent manner.

#### 3.5.12. Narrative Synthesis on the Effect of SFN on Cell Invasion and Cell Migration

Jeong and colleagues, 2017 [28] illustrated that the migration activity of U-2 OS cells, induced by a potent tumour promoter (12-O-tetradecanoylphorbol-13-acetate [TPA]), was significantly reduced following a 24 h treatment with 10 μM SFN. These results suggest that SFN treatment compromised the in vitro migrative abilities of the human OSA cell line U-2 OS (Appendix C, Table A8).

#### 3.5.13. Effect of SFN on Tumour Volume and Weight (Narrative Synthesis Only)

Matsui and colleagues, 2007 [17], were the first to report the effects of SFN in a mouse OSA (LM8) xenograft model. The authors showed that intraperitoneal administration of SFN at doses of 5 mg/week and 10 mg/week, for the period of 4 weeks significantly reduced the mean tumour volume/mouse (mm^3^) compared to controls, by a factor of 4 and 4.4, respectively (Appendix C, Table A9). Eighteen years later, Zou and colleagues, 2025 [31] also studied the dose-dependent antitumour effects of SFN in a different mouse OSA (143B) xenograft model. They showed that intraperitoneal administration of SFN at 25 mg/kg or 50 mg/kg every two days, for a period of 18 days significantly reduced the mean tumour volume (mm^3^) by a factor of 1.9 and 2.7, respectively, compared to saline-treated mice. The tumour weight (g) was also significantly reduced by a factor of 1.7 and 2.5, following treatment with SFN at 25 mg/kg and 50 mg/kg, respectively. These findings suggest that SFN dose-dependently inhibited the in vivo proliferation of OSA cells.

## 4. Discussion

This review and meta-analysis present an investigation into the preclinical evidence for the effect of SFN as a therapeutic agent for OSA, in particular in relation to the proliferation of cancer cells. Data from ten preclinical studies using a mix of human (seven studies) and non-human (one study canine, one study murine, and one murine and human study) cell lines was synthesized narratively and quantitatively through meta-analysis. Overall, the results suggest that SNF might be of use in the treatment of OSA in humans. Although the specific mechanism through which SNF affects OSA outcomes is still under investigation, several preclinical and clinical studies conducted in other forms of cancer [10] suggest that SNF has an inhibitory effect on cancer cell growth and can also be used as adjunctive treatment in chemosensitization.

At this preclinical stage, and given the studies reviewed here, the focus has been mainly on the effect on cancer cell growth and proliferation. One of the primary actions of SFN is its ability to induce apoptosis in cancer cells. One of the reviewed studies [19] suggests that SFN enhances the sensitivity of OSA cells to apoptosis through the upregulation of the death receptor 5 (DR5) pathway. This modulation sensitizes the cancer cells to TNF-related apoptosis-inducing ligand (TRAIL)-mediated cell death, facilitating a greater apoptotic response without affecting normal cells. This specificity towards cancer cells is particularly advantageous given that it potentially minimizes the cytotoxic impact on healthy tissues. SFN has also been found to arrest the cell cycle at the G2/M phase in OSA cells, halting their uncontrolled proliferation. Three studies reviewed here [17,18,28] suggest that the effect is achieved through the downregulation of cyclins and cyclin-dependent kinases (CDKs) and the concurrent upregulation of CDK inhibitors like p21. Therefore, by disrupting the cell cycle, SFN effectively reduces the proliferation rate of OSA cells. In addition to affecting cell cycle regulation and apoptosis, SFN seems to inhibit critical signaling pathways in OSA by downregulating the PI3K/AKT and MAPK pathways, both of which are frequently overactivated in these cancer cells, by inhibiting the phosphorylation of Akt and ERK, key regulators of cell survival and proliferation, and therefore promoting cell viability and growth [20]. Inhibition of these pathways can lead to reduced cell proliferation and increased apoptotic rates. SFN also impacts the tumour microenvironment, particularly through its anti-angiogenic properties and inhibition of matrix metalloproteinases (MMPs), such as MMP-9. These effects contribute to decreasing tumour invasiveness and metastatic potential, as seen in various cancer cell models [28].

Another study suggests that SFN seems to cause DNA damage and mitotic abnormalities in OSA cells, leading to cell cycle arrest at the G2/M phase. This arrest is part of the broader mechanism by which SFN limits cell proliferation and facilitates apoptotic pathways [7].

SFN also seems to induce oxidative stress in OSA cells by increasing ROS levels. SFN impairs glutathione recycling by inhibiting enzymes such as glutathione reductase and glutathione peroxidase. This inhibition contributes to the accumulation of oxidative stress, further promoting the pro-apoptotic effects of SFN. One of the reviewed studies revealed that this specific apoptotic process seems to be independent of p53, a tumour suppressor gene often mutated in OSA [27]. In the latest reviewed study, SFN in OS cells appears to directly target the p62 protein and promote the lysosomal degradation pathway of the cystine transporter SLC7A11 by enhancing p62/SLC7A11 protein–protein interaction. This, in turn, disrupts redox homeostasis, promotes ROS production and lipid peroxidation, and ultimately induces ferroptosis [31].

One interesting finding from this review is that overall, the effects of SFN on OSA cells, independent of the pathway studied, seem to be both dose and exposure-dependent, with increased doses and time of exposure contributing to better outcomes in OSA. Some exceptions are noted in the results (e.g., lack of antioxidant activity); however, the overall pooled analysis seems to favor the assumptions that SFN is beneficial in OSA. This evidence is congruent with a recent review that addressed the effect of SFN across several types of cancer [10]. For example, TRAIL-mediated cell death due to exposure to SFN was also reported in bladder cancer [32], prostate cancer [33], and hepatoma (primary liver cancer) [34]. SFN-induced G2/M phase cell cycle arrest has also been reported in ovarian [35] lung [36] and colorectal cancers [37].

There are several limitations to this review and meta-analysis, the main one being the very low number of studies synthesized and the heterogeneity of the targeted processes studied. This is reflective of the current infant stage of research on the effect of SFN on OSA, where different causal hypotheses for the observed effects are still under investigation. Another point of heterogeneity observed was the fact that some of the observed effects come from studies that did not use human cell lines, therefore the translational potential of those findings might be limited. Moving forward in the field of SFN for OSA, we believe some steps would be essential in reducing heterogeneity and increasing the potential for real-life applications.

First, key mediating processes of the effect of SFN on OSA should be further studied and replicated. TRAIL-mediated cell death and G2/M stage proliferation arrest seem to be the most promising areas to be replicated. Preliminary human trials are also essential to establish any translational or real-life application potential. Several human trials have already been conducted with prostate, breast, pancreatic cancers, and melanoma with overall good results. Given the overall safety profile of SFN as a nutraceutical dietary component, these could be easily implemented in multiple baseline single-case designs with relatively low risk for participants. However, understanding its interaction with other dietary components is essential, as it may either potentiate or reduce the observed effects of SFN. For example, SFN was shown to interact antagonistically with furosemide, verapamil, and ketoprofen [38]; therefore, caution is needed in the introduction of this chemopreventative compound. Another possible interaction regards the potential to compromise immunotherapeutic interventions, as SFN has been observed to suppress T cell-mediated immune responses [39]. Due to its unstable chemical structure, SFN is highly sensitive to both oxygen and pH and is therefore rapidly degraded in response to heat and light [40]. Not surprisingly, the pharmacokinetics of SFN are highly variable and inconsistent, making it difficult to maintain an efficacious concentration for an adequate duration. Numerous clinical studies indicate that SFN reaches only low micromolar peak plasma concentrations and exhibits a short biological half-life in clinical trial settings [41,42,43,44]. Low bioavailability challenges such as these, coupled with difficulties in mass producing and storing SFN [45], bring questions on the scalability of SFN as a feasible adjuvant intervention in OSA and other cancers.

## 5. Conclusions

In conclusion, this review has comprehensively examined the evidence for the use of SFN in OSA at a preclinical stage on outcomes primarily related to cell apoptosis and proliferation. Although the overall evidence seems to favor the use of SFN for OSA, the heterogeneity of studies and lack of human trials curtail the possibility of making a stronger recommendation. We propose a pathway for the continuation of this line of research that will reduce some of the bias in evidence and promote a translational approach to this topic.

## Figures and Tables

**Table 1:** Characteristics of included studies.

Study, Year	Osteosarcoma Cell Line (s)	Intervention	Reported Cancer Relevant Outcomes	Study Results	Funding Source
Ferreira de Oliveira et al., 2014a [27]	Human: MG-63	In vitro: 0, 5, 10, and 20 μΜ SFN	Cell viability Apoptosis Caspase-3 activity Total antioxidant activity GSH levels ROS formation	Cell viability loss and increase in apoptosis Increase in caspase-3 activity ROS induction and decrease in antioxidant enzymes like SOD, GPx, and GR Decrease in intracellular GSH levels	Portuguese Foundation for Science and Technology
Ferreira de Oliveira et al., 2014b [7]	Human: MG-63	In vitro: 0, 5, 10, and 20 μM SFN	Cell viability Apoptosis Cell cycle distribution DNA damage quantification Cytoskeletal disruption	Dose- and time-dependent cytotoxicity Cell cycle arrest at the G2/M phase Alterations in the cytoskeletal network Increase in DNA fragmentation Downregulation of Chk1 and Cdc25C while increased CDK1 expression	Portuguese Foundation for Science and Technology
Jeong et al., 2017 [28]	Human: U-2 OS	In vitro: 10 µM SFN	Cell invasion Cell migration MMP-9 gene expression Angiogenesis	Reduction in TPA-induced MMP-9 activity and protein expression in U-2 OS cells Inhibition of U-2 OS cell migration Suppression of the transcriptional activity of AP-1 and NF-κB in the MMP-9 promoter Reduction in tumour cell invasion and migration	National Research Foundation of Korea, Ministry of Science, ICT & Future Planning, Korea Research Institute of Bioscience & Biotechnology
Kim et al., 2011 [18]	Human: U-2 OS, MG-63	In vitro: 0, 1, 5, 10, and 20 µM SFN	Cell proliferation Cell cycle distribution Apoptosis DNA fragmentation	Inhibition of cell viability in U-2 OS and MG-63 cells G2/M phase arrest in U-2 OS cells, with a notable increase in the % of cells in the G2/M phase Increase in annexin V+/PI- cells in U-2 OS cells Downregulation of the expression of cyclins A and B1, as well as CDKs 1 and 2	Chonbuk National University Hospital Research Institute of Clinical Medicine and research funds
Li et al., 2016 [29]	Human; OS-732, MG-63	In vitro: 0, 1, 5, 10, 15, and 20 µM SFN	Cell viability Apoptosis Cell cycle distribution	Dose-dependent decrease in cell survival Increase in G1-phase arrest Induction of apoptosis SFN and Cisplatin combination reduces the expression of cyclin E and D and activates the tumour suppressor p53-p21 pathway	No information
Matsui et al., 2006 [19]	Human: Saos2, MG-63	In vitro: 0–40 µM SFN	Apoptosis Cell viability	Reduction in cell viability in Saos2 cells in a dose-dependent manner Upregulation of the expression of DR5 Combination of SFN and TRAIL triggered the apoptotic caspase cascade	Ministry of Education, Culture, Sports, Science and Technology of Japan.
Matsui et al., 2007 [17]	Human: MG-63 Murine: LM8	In vitro: 0–20 μΜ SFN In vivo: BALB/C mice, i.p. injection, 5 mg/week and 10 mg/week (for 4 weeks)	Cell viability Cell cycle distribution Apoptosis In vivo tumour growth	Dose-dependent decrease in cell growth Induction of G2/M phase arrest through the p53-independent activation of p21WAF1/CIP1 Induction of apoptosis in LM8 cells Inhibition of LM8 xenograft tumour growth in vivo	Ministry of Education, Culture, Sports, Science and Technology of Japan & Takeda Science Foundation
Rizzo et al., 2017 [30]	Canine: OS 2.4, HMPOS, D17	In vitro: 0.8 to 100 μΜ SFN	Cell viability Cell invasion Cell cycle distribution ROS formation	Increased viability of D17 and OS 2.4 cells at conc. 0.8–6.25 μM and decreased viability at conc. 25–100 μM Decreased viability of HMPOS cells (3.13–100 μM) Reduction in cell invasion Failure to prove that SFN could induce cell death (up to 50 μM) Substantial decrease in the phosphorylation of focal adhesion kinase (FAK) in D17 and OS 2.4 cells OS 2.4 cells showed a significant decrease in the G1/0 phase	No information
Sawai et al., 2013 [20]	Murine: LM8	In vitro: 0, 2.5, 5, 10, 15, and 20 µM SFN	Cell cycle distribution Apoptosis Cell viability	Increase in the population of cells in the G2/M phase Inhibition of cell growth dose-dependently Combination of SFN and radiation effectively induced nuclear fragmentation and apoptosis Induction of apoptosis through G2/M-phase arrest and by inhibiting ERK and Akt activation	KAKENHI (Grant-in-Aid for Scientific Research)
Zou et al., 2025 [31]	Human: 143B, SJSA-1	In vitro: 0–20 μΜ SFN In vivo: BALB/C mice, i.p. injection, 25 mg/kg or 50 mg/kg every two days (for 18 days)	Cell viability Colony formation ROS formation GSH levels Ferroptosis induction In vivo tumour growth	Dose- and time-dependent inhibition of cell viability in 143B and SJSA-1 cells Reduced colony formation Elevated levels of ROS and increased lipid peroxidation along with reduction in GSH levels Induction of ferroptosis through downregulation of SLC7A11 Dose dependent reduction in tumour growth in subcutaneous tumour OSA model In vivo induction of ferroptosis in tumour tissues by downregulation of Ki67, SLC7A11 and GPX4 without causing systemic toxicity	Guangdong Basic and Applied Basic Research Foundation, National Natural Science Foundation of China, Science and Technology Projects in Guangzhou, Qilu Sanitation and Health LeadingTalent Cultivation Project and the Medical Joint Fund of Jinan University

Abbreviations: SFN, sulforaphane; GSH, glutathione; ROS, reactive oxygen species; TPA, 12-O-tetradecanoylphorbol-13-acetate; PI, propidium iodide; DR5, death receptor 5; TRAIL, tumour necrosis factor (TNF) related apoptosis-inducing ligand; conc., concentration(s); i.p., intraperitoneal; ERK, extracellular signal-regulated kinase; OSA, osteosarcoma; Ki67, marker of proliferation Kiel 67; SLC7A11, Solute Carrier Family 7 Member 11; GPX4, Glutathione peroxidase 4.

## Data Availability

No new data were created or analyzed in this study.

## Supplementary Materials

The following supporting information can be downloaded at: https://www.mdpi.com/article/10.3390/biomedicines13051048/s1, Table S1: PRISMA 2020 Checklist; Table S2: PRISMA 2020 for Abstracts Checklist; Table S3: Search strategy and keywords for Medline and Embase; Table S4: Search strategy and keywords for Web of Science; Table S5: Breakdown of how WebPlotDigitizer Version 4.8 works; Table S6: Details of the study excluded in full-text stage.

## Abbreviations

CDKs, cyclin-dependent kinases; CI, confidence intervals; Conc., concentration(s); DR5, death receptor 5; ERK, extracellular signal-regulated kinase; GPX4, glutathione peroxidase 4; GRADE, grading of recommendations assessment, development, and evaluation; GSH, glutathione; i.p., intraperitoneal; INPLASY, International Platform of Registered Systematic Review and Meta-Analysis Protocols; ITCs, isothiocyanates; IV, inverse-variance weighting; Ki67, marker of proliferation Kiel 67; MeSH, medical subject heading; MMPs, matrix metalloproteinases; OHAT, Office of Health Assessment and Translation; OSA, osteosarcoma; PI, propidium iodide; PRISMA, Preferred Reporting Items for Systematic Review and Meta-Analysis; RevMan, Review Manager; ROS, reactive oxygen species; SD, standard deviation; SEM, standard error of the mean; SFN, sulforaphane; SLC7A11, Solute Carrier Family 7 Member 11; Std., standardized; TNF, tumour necrosis factor; TPA, 12-O-tetradecanoylphorbol-13-acetate; TRAIL, TNF-related apoptosis-inducing ligand.

## Appendix B

**Table A1:** Summary of meta-analysis of the impact of sulforaphane (SFN) on in vitro cancer outcomes.

Outcome	Studies Included	Cell line (Human/Mouse)	SFN Concentration	Treatment Duration (Hours)	Subgroup (std.) Mean Difference (95% CI)	Overall (std.) Mean Difference (95% CI)	Statistical Method	Test for Heterogeneity	Test for Overall Effect
Cell viability	Kim et al., 2011 [18]; Ferreira de Oliveira et al., 2014b [7]; Li et al., 2016 [29]	MG-63 (human)	5 μΜ	24 48 72	−3.13 (−18.02, 11.75) −38.93 (−78.30, 0.45) ***−23.89 (−31.41, −16.37)***	−26.05 (−53.30, 1.19)	Mean difference, IV, Random effects, 95% CI	Chi^2^ = 130.38, df = 3, *p* < 0.00001, I^2^ = 98%	Z = 1.87, *p* = 0.06
		MG-63 (human)	10 μΜ	24 48 72	−1.69 (−10.97, 7.6) ***−69.14 (−70.73, −67.56)*** ***−36.50 (−42.98, −30.01)***	***−41.40 (−73.50, −9.31)***	Mean difference, IV, Random effects, 95% CI	Chi^2^ = 279.26, df = 3, *p* < 0.00001, I^2^ = 99%	***Z = 2.53, p = 0.01***
	Kim et al., 2011 [18]; Jeong et al., 2017 [28]	U-2 OS (human)	5 μΜ	24 48	−4.09 (−20.41, 12.22) ***−8.38 (−13.83, −2.92)***	***−7.95 (−13.12, −2.78)***	Mean difference, IV, Fixed effects, 95% CI	Chi^2^ = 0.24, df = 1, *p* = 0.63, I^2^ = 0%	***Z = 3.01, p = 0.003***
		U-2 OS (human)	10 μΜ	24 48	−2.24 (−12.62, 8.14) ***−26.22 (−29.30, −23.13)***	−14.76 (−38.23, 8.71)	Mean difference, IV, Random effects, 95% CI	Chi^2^ = 18.82, df = 1, *p* < 0.0001, I^2^ = 95%	Z = 1.23, *p* = 0.22
Cell cycle distribution: G0/G1 phase	Kim et al., 2011 [18]; Ferreira de Oliveira et al., 2014b [7]	MG-63 (human)	5 μΜ	24 48	***−8.98 (−16.38, −1.58)*** ***−15.55 (−26.64, −4.46)***	***−10.39 (−16.96, −3.83)***	Mean difference, IV, Random effects, 95% CI	Chi^2^ = 5.12, df = 2, *p* = 0.08, I^2^ = 61%	***Z = 3.10, p = 0.002***
		MG-63 (human)	10 μΜ	24 48	***−19.93 (−23.00, −16.86)*** ***−31.10 (−55.17, −7.02)***	***−20.11 (−23.15, −17.06)***	Mean difference, IV, Fixed effects, 95% CI	Chi^2^ = 0.89, df = 2, *p* = 0.64, I^2^ = 0%	***Z = 12.94, p < 0.00001***
Cell cycle distribution: S phase	Kim et al., 2011 [18]; Ferreira de Oliveira et al., 2014b [7]	MG-63 (human)	5 μΜ	24 48	0.78 (−9.38, 10.94) ***6.01 (2.01, 10.01)***	2.54 (−5.19, 10.27)	Mean difference, IV, Random effects, 95% CI	Chi^2^ = 20.18, df = 2, *p* < 0.0001, I^2^ = 90%	Z = 0.64, *p* = 0.52
		MG-63 (human)	10 μΜ	24 48	−1.19 (−8.32, 5.95) −6.71 (−0.44, 13.87)	0.93 (−5.03, 6.89)	Mean difference, IV, Random effects, 95% CI	Chi^2^ = 9.67, df = 2, *p* = 0.008, I^2^ = 79%	Z = 0.31, *p* = 0.76
Cell cycle distribution: G2/M phase	Kim et al., 2011 [18]; Ferreira de Oliveira et al., 2014b [7]	MG-63 (human)	5 μΜ	24 48	***10.39 (6.53, 14.24)*** ***10.25 (1.51, 18.98)***	***10.36 (6.84, 13.89)***	Mean difference, IV, Fixed effects, 95% CI	Chi^2^ = 0.36, df = 2, *p* = 0.84, I^2^ = 0%	***Z = 5.76, p < 0.00001***
		MG-63 (human)	10 μΜ	24 48	***16.61 (12.91, 20.31)*** ***24.03 (7.86, 40.20)***	***16.98 (13.37, 20.59)***	Mean difference, IV, Fixed effects, 95% CI	Chi^2^ = 3.15, df = 2, *p* = 0.21, I^2^ = 36%	***Z = 9.22, p < 0.00001***
Apoptosis indices (Sub-G1 phase & Active caspase-3)	Matsui et al., 2006 [19]; Ferreira de Oliveira et al., 2014a [27]	MG-63 (human)	20 μΜ	24 (Sub-G1) 48 (Active caspase-3)	3.34 (−0.26, 6.94) 1.26 (−6.13, 8.66)	2.94 (−0.30, 6.18)	Std. mean difference, IV, Fixed effects, 95% CI	Chi^2^ = 0.25, df = 1, *p* = 0.62, I^2^ = 0%	Z = 1.78, *p* = 0.07
Trypan blue dye exclusion test	Matsui et al., 2007 [17]; Sawai et al., 2013 [20]	LM8 (murine)	5 μΜ 10 μΜ 20 μΜ	24	***−3.16 (−5.88, −0.45)*** ***−5.21 (−9.42, −0.99)*** ***−9.49 (−16.61, −2.37)***	***−4.30 (−6.47, −2.12)***	Std. mean difference, IV, Fixed effects, 95% CI	Chi^2^ = 7.49, df = 5, *p* = 0.19, I^2^ = 33%	***Z = 3.87, p = 0.0001***
Antioxidant activity (total antioxidant activity & intracellular glutathione levels)	Ferreira de Oliveira et al., 2014a [27]	MG-63 (human)	5 μΜ	24 (total antioxidant activity) 24 (intracellular glutathione levels)	−0.84 (−2.63, 0.96) 0.81 (−4.17, 5.78)	−0.65 (−2.33, 1.04)	Std. mean difference, IV, Fixed effects, 95% CI	Chi^2^ = 0.37, df = 1, *p* = 0.54, I^2^ = 0%	Z = 0.75, *p* = 0.45
	Ferreira de Oliveira et al., 2014a [27]	MG-63 (human)	10 μΜ	24 (total antioxidant activity) 24 (intracellular glutathione levels)	−1.52 (−3.70, 0.65) 0.10 (−1.94, 2.13)	−0.66 (−2.15, 0.83)	Std. mean difference, IV, Fixed effects, 95% CI	Chi^2^ = 1.14, df = 1, *p* = 0.29, I^2^ = 12%	Z = 0.87, *p* = 0.38
		MG-63 (human)	20 μΜ	24 (total antioxidant activity) 24 (intracellular glutathione levels)	−3.36 (−6.99, 0.26) −1.65 (−11.18, 7.88)	−3.15 (−6.53, 0.24)	Std. mean difference, IV, Fixed effects, 95% CI	Chi^2^ = 0.11, df = 1, *p* = 0.74, I^2^ = 0%	Z = 1.82, *p* = 0.07
Antioxidant activity (total antioxidant activity & intracellular glutathione levels)	Ferreira de Oliveira et al., 2014b [7]	MG-63 (human)	5 μΜ	48 (total antioxidant activity) 48 (intracellular glutathione levels)	−0.18 (−1.79, 1.43) 2.17 (−10.26, 14.59)	−0.14 (−1.74, 1.45)	Std. mean difference, IV, Fixed effects, 95% CI	Chi^2^ = 0.14, df = 1, *p* = 0.71, I^2^ = 0%	Z = 0.18, *p* = 0.86
		MG-63 (human)	10 μΜ	48 (total antioxidant activity) 48 (intracellular glutathione levels)	−0.21 (−1.82, 1.41) 2.29 (−10.79, 15.37)	−0.17 (−1.77, 1.43)	Std. mean difference, IV, Fixed effects, 95% CI	Chi^2^ = 0.14, df = 1, *p* = 0.71, I^2^ = 0%	Z = 0.21, *p* = 0.84
Cellinvasion capabilities	Rizzo et al., 2017 [30]	D17, OS 2.4, HMPOS (canine)	10 μΜ	24	--------------------------	−25.21 (−42.01, −8.42)	Mean difference, IV, effects, 95% CI	Chi^2^ = 2.92, df = 2, *p* = 0.23, I^2^ = 31%	Z = 2.94, *p* = 0.003,

Std., standardized; IV, inverse-variance weighting. In bold and italics are statistically significant results.

## Appendix C

**Table A2:** Data for narrative synthesis of the effect of SFN on cell viability and colony formation.

Viability of Human Osteosarcoma Cell Lines (Mean ± SD)						
Study	Cell Line	Duration (Hours)	Vehicle	SFN 5 μM	SFN 10 μM	SFN 20 μM
Li et al., 2016 [29]	OS-732	72	100 ± 0.84	82.25 ± 6.29 **	69.21 ± 4.94 **	48.99 ± 4.94 ***
Zou et al., 2025 [31]	143B	24	100 ± 4.46	85.89 ± 1.78	57.05 ± 2.33	33.18 ± 2.26
	SJSA-1	24	100 ± 1.97	81.24 ± 4.25	66.98 ± 3.90	41.24 ± 3.22
	143B	48	100 ± 1.09	56.78 ± 3.69	18.72 ± 1.44	11.96 ± 1.68
	SJSA-1	48	100 ± 0.78	51.56 ± 2.26	42.05 ± 4.22	23.02 ± 1.31
**Viability of D17, OS 2.4 and HMPOS for 48 h (Mean ± SD)**						
**Study**	**Cell Line**	**Duration (Hours)**	**Vehicle**	**SFN 6.25** **μM**	**SFN 12.5** **μM**	**SFN 25** **μM**
Rizzo et al., 2017 [30]	D17	48	100 ± 6.42	125.99 ± 11.01 * (↑)	88.69 ± 13.45	58.10 ± 8.57 * (↓)
	OS 2.4	48	100 ± 6.13	130.06 ± 14.11 * (↑)	69.94 ± 3.68	36.81 ± 3.06 * (↓)
	HMPOS	48	100 ± 3.05	65.24 ± 12.19 * (↓)	56.10 ± 4.88 * (↓)	64.02 ± 10.36 * (↓)
**Colony Formation in 143B and SJSA-1 Cell Cultures**						
**Study**	**Cell Line**	**Vehicle**	**SFN 1.25** **μM**		**SFN 2.5** **μM**	**SFN 5** **μM**
Zou et al., 2025 [31]	143B	98.36 ± 8.61	68.85 ± 7.99 *		51.64 ± 4.92 **	2.46 ± 3.69 **
	SJSA-1	97.13 ± 14.14	76.84 ± 15.37		55.33 ± 15.98 *	12.91 ± 7.99 **

↑ indicates increase; ↓ indicates decrease. * *p* < 0.05; ** *p* < 0.01; *** *p* < 0.001.

**Table A3:** Data for narrative synthesis of the effect of SFN on cell cycle distribution.

	Cell Cycle Distribution of OS-732 After 72 h Treatment with Vehicle DMSO			
Study	Cell Line	G0/G1	S	G2/M
Li et al., 2016 [29]	OS-732	38.3 ± 2.13	14.47 ± 1.27	46.8 ± 2.56
	**Cell cycle distribution of OS-732 after 72 h treatment with 15** **μM SFN**			
	**Cell line**	**G0/G1**	**S**	**G2/M**
	OS-732	54.89 ± 2.13	11.07 ± 1.27	33.61 ± 2.56
	**Cell cycle distribution of MG-63 after 72 h treatment with vehicle DMSO**			
**Study**	**Cell line**	**G0/G1**	**S**	**G2/M**
Li et al., 2016 [29]	MG-63	43.27 ± 1.85	15.4 ± 1.23	42.04 ± 2.15
	**Cell cycle distribution of MG-63 after 72 h treatment with 15** **μM SFN**			
	**Cell line**	**G0/G1**	**S**	**G2/M**
	MG-63	59.23 ± 0.62	8.29 ± 0.92	31.92 ± 2.45
	**Cell cycle distribution of D17 after 48 h treatment with vehicle DMSO**			
**Study**	**Cell line**	**G0/G1**	**S**	**G2/M**
Rizzo et al., 2017 [30]	D17	56.6 ± 1.6	16.3 ± 0.7	26.4 ± 1.4
	**Cell cycle distribution of D17 after 48 h treatment with 20** **μM SFN (* *p* < 0.05 from vehicle)**			
	**Cell line**	**G0/G1**	**S**	**G2/M**
	D17	55.0 ± 1.7	12.2 ± 0.3 *	32.5 ± 0.9 *
	**Cell cycle distribution of OS 2.4 after 48 h treatment with vehicle DMSO**			
**Study**	**Cell line**	**G0/G1**	**S**	**G2/M**
Rizzo et al., 2017 [30]	OS 2.4	73.0 ± 1.4	7.2 ± 0.2	19.5 ± 1.4
	**Cell cycle distribution of OS 2.4 after 48 h treatment with 20** **μM SFN (* *p* < 0.05 from vehicle)**			
	**Cell line**	**G0/G1**	**S**	**G2/M**
	OS 2.4	54.1 ± 1.3 *	13.0 ± 0.2 *	31.8 ± 1.6 *
	**Cell cycle distribution of HMPOS after 48 h treatment with vehicle DMSO**			
**Study**	**Cell line**	**G0/G1**	**S**	**G2/M**
Rizzo et al., 2017 [30]	HMPOS	75.5 ± 1.1	8.0 ± 0.3	16.4 ± 1.0
	**Cell cycle distribution of HMPOS after 48 h treatment with 20** **μM SFN (* *p* < 0.05 from vehicle)**			
	**Cell line**	**G0/G1**	**S**	**G2/M**
	HMPOS	70.1 ± 1.8	6.8 ± 0.8	23.3 ± 1.4*
	**Cell cycle distribution of LM8 after 24 h treatment with vehicle DMSO**			
**Study**	**Cell line**	**G1**	**S**	**G2/M**
Matsui et al., 2007 ^+^ [17]	LM8	31.10	55.98	12.92
	**Cell cycle distribution of LM8 after 24 h treatment with 20** **μM SFN**			
	**Cell line**	**G1**	**S**	**G2/M**
	LM8	15.55	51.91	32.54

^+^ No SD values provided.

**Table A4:** Data for narrative synthesis of the effect of SFN on the fraction of LM8 cells in the Sub-G1 phase.

Sub-G1% of Cells After 24 h Treatment with Vehicle or SFN (Mean ± SD)						
Study	Cell Line	Vehicle	2.5 μM	5 μM	10 μM	15 μM
Sawai et al., 2013 [20]	LM8	0.67 ± 0.005	0.95 ± 0.003	1.52 ± 0.004	1.90 ± 0.10	7.03 ± 1.81

**Table A5:** Data for narrative synthesis of the effect of SFN on the trypan blue dye exclusion test.

Trypan Blue Dye Exclusion Test for MG-63 Cells with SFN for 24 h (×10^4^; Mean ± SD) (* *p* < 0.05)					
Study	Cell Line	Vehicle	SFN 5 μM	SFN 10 μM	SFN 20 μM
Matsui et al., 2007 [17]	MG-63	3.55 ± 0.47	3.08 ± 0.27	2.53 ± 0.16 *	1.33 ± 0.27 *
**Trypan blue dye exclusion test for MG-63 cells with SFN for 48 h (×10^4^; mean ± SD) (* *p* < 0.05)**					
**Study**	**Cell line**	**Vehicle**	**SFN 5** **μM**	**SFN 10** **μM**	**SFN 20** **μM**
Matsui et al., 2007 [17]	MG-63	10.97 ± 1.01	8.13 ± 0.62 *	4.36 ± 0.62 *	1.21 ± 0.31 *
**Trypan blue dye exclusion test for D17, OS 2.4 and HMPOS cells with 20** **μM SFN for 48 h (% trypan blue positive cells, i.e., non-viable cells; mean ± SD) (* *p* < 0.05)**					
**Study**	**Cell line**		**Vehicle**	**SFN 20** **μM**	
Rizzo et al., 2017 [30]	D17		5 ± 3	11 ± 3	
	OS 2.4		28 ± 3	5 ± 5 * (↓)	
	HMPOS		7 ± 3	7 ± 5	

↓ indicates decrease.

**Table A6:** Data for narrative synthesis of the effect of SFN on the percentage of apoptotic cells.

% Apoptotic OS-732 Cells with 15 μΜ SFN for 72 h (Mean ± SD) (* *p* < 0.001)				
Study	Cell Line	Vehicle	SFN 15 μM	
Li et al., 2016 [29] 72 h	OS-732	6.14 ± 1.62	23.28 ± 2.75 *	
**% apoptotic ΜG-63 cells with SFN (mean ± SD) (* *p* < 0.05)**				
**Study**	**Cell line**	**Vehicle**	**SFN 5** **μM**	**SFN 10** **μM**
Ferreira de Oliveira et al., 2014b [7] 24 h	MG-63	9.12 ± 1.83	11.23 ± 3.05	14.04 ± 2.44 *
Ferreira de Oliveira et al., 2014b [7] 48 h		8.77 ± 1.78	10.31 ± 1.78	26.77 ± 3.56 *

**Table A7:** Data for narrative synthesis of the effect of SFN on reactive oxygen species formation.

ROS Accumulation (Median Fluorescent Intensity) MG-63 Cells with SFN (Mean ± SD) (* *p* < 0.05)						
Study		Cell Line	Vehicle	SFN 5 μM	SFN 10 μM	SFN 20 μM
Ferreira de Oliveira et al., 2014a [27] SFN (for 24 h)		MG-63	0.92 ± 0.24	1.27 ± 0.19	3.22 ± 0.61 *	3.84 ± 0.21 *
Ferreira de Oliveira et al., 2014a [27] SFN (for 48 h)			0.63 ± 0.09	1.02 ± 0.16 *	4.22 ± 0.23 *	4.00 ± 0.26 *
**ROS accumulation/Dihydrorhodamine 123 fluorescence assay (mean ± SD) (* *p* < 0.05)**						
**Study**		**Cell line**	**Vehicle**		**SFN 20** **μM**	
Rizzo et al., 2017 [30]		D17	652.46 ± 72.13		773.77 ± 144.26	
		OS 2.4	186.89 ± 22.95		337.71 ± 16.39 *	
		HMPOS	459.02 ± 39.34		62.30 ± 9.11 *	
**ROS accumulation (relative ROS fluorescence) (mean ± SD) (* *p* < 0.05; ** *p* < 0.01)**						
**Study**	**Cell line**	**Vehicle**	**SFN 5** **μM**		**SFN 10** **μM**	
Zou et al. [31]	143B	100 ± 0.34	116.16 ± 5.05 *		137.37 ± 12.12 **	
	SJSA-1	100 ± 0.42	145.46 ± 8.08 **		150.50 ± 7.08 **	

**Table A8:** Data for narrative synthesis of the effect of SFN on cell migration.

Migration Activity in U-2OS Cells Treated with SFN 10 μM and 12-O-Tetradecanoylphorbol-13-Acetate (TPA) (% of Control, Mean ± SD) (* *p* < 0.05)			
Study	Cell Line	Vehicle + TPA	SFN 10 μM + TPA
Jeong et al., 2017 [28]	U-2 OS	100 ± 9.62	49.26 ± 5.77 *

**Table A9:** Data for narrative synthesis of the effect of SFN on tumour volume and weight.

Tumour Volume from LM8 Cells Following Treatment with SFN for 4 Weeks (mean ± SD) (* *p* < 0.05)				
Study	Parameter	Vehicle	SFN 5 mg/week	SFN 10 mg/week
Matsui et al., 2007 [17]	Tumour volume (cm^3^)	906.82 ± 245.45	225.00 ± 81.82 *	204.55 ± 115.91 *
**Tumour volume from 143B cells following treatment with SFN for 18 days (mean ± SD) (* *p* < 0.05; ** *p* < 0.01)**				
**Study**	**Parameter**	**Vehicle**	**SFN 25** **mg/kg**	**SFN 50** **mg/kg**
Zou et al., 2025 [31]	Tumour volume (mm^3^)	1364 ± 519.29	734 ± 240.05 *	511 ± 159.22 **
Zou et al., 2025 [31]	Weight (g)	0.823 ± 0.326	0.483 ± 0.073 *	0.323 ± 0.166 **
